# Partial Discharge Signal Pattern Recognition of Composite Insulation Defects in Cross-Linked Polyethylene Cables

**DOI:** 10.3390/s24113460

**Published:** 2024-05-27

**Authors:** Chunxu Qin, Xiaokai Zhu, Pengfei Zhu, Wenjie Lin, Liqiang Liu, Chuanqiang Che, Huijuan Liang, Huichun Hua

**Affiliations:** 1 Engineering Research Center of Large Energy Storage Technology, Ministry of Education, Inner Mongolia University of Technology, Hohhot 010051, China; qincx@imut.edu.cn (C.Q.); 18390217716@163.com (X.Z.); islinwj@126.com (W.L.); huijuanl01@163.com (H.L.); 2 Hebei Key Laboratory of Physics and Energy Technology, North China Electric Power University, Baoding 071000, China; huahuichun@ncepu.edu.cn; 3 Xuchang Power Supply Company, State Grid Henan Electric Power Company, Xuchang 461000, China; 13298265779@163.com; 4 Inner Mongolia Electric Power Scientific Research Institute, Hohhot 010020, China; imccq@126.com

**Keywords:** XLPE cables, partial discharge, composite defect, snake optimizer–support vector machine, pattern recognition

## Abstract

To investigate the pattern recognition of complex defect types in XLPE (cross-linked polyethylene) cable partial discharges and analyze the effectiveness of identifying partial discharge signal patterns, this study employs the variational mode decomposition (VMD) algorithm alongside entropy theories such as power spectrum entropy, fuzzy entropy, and permutation entropy for feature extraction from partial discharge signals of composite insulation defects. The mean power spectrum entropy (PS), mean fuzzy entropy (FU), mean permutation entropy (PE), as well as the permutation entropy values of IMF2 and IMF13 (Pe) are selected as the characteristic quantities for four categories of partial discharge signals associated with composite defects. Six hundred samples are selected from the partial discharge signals of each type of compound defect, amounting to a total of 2400 samples for the four types of compound defects combined. Each sample comprises five feature values, which are compiled into a dataset. A Snake Optimization Algorithm-optimized Support Vector Machine (SO-SVM) model is designed and trained, using the extracted features from cable partial discharge datasets as case examples for recognizing cable partial discharge signals. The identification outcomes from the SO-SVM model are then compared with those from conventional learning models. The results demonstrate that for partial discharge signals of XLPE cable composite insulation defects, the SO-SVM model yields better identification results than traditional learning models. In terms of recognition accuracy, for scratch and water ingress defects, SO-SVM improves by 14.00% over BP (Back Propagation) neural networks, by 5.66% over GA-BP (Genetic Algorithm–Back Propagation), and by 12.50% over SVM (support vector machine). For defects involving metal impurities and scratches, SO-SVM improves by 13.39% over BP, 9.34% over GA-BP, and 12.56% over SVM. For defects with metal impurities and water ingress, SO-SVM shows enhancements of 13.80% over BP, 9.47% over GA-BP, and 13.97% over SVM. Lastly, for defects combining metal impurities, water ingress, and scratches, SO-SVM registers increases of 11.90% over BP, 9.59% over GA-BP, and 12.05% over SVM.

## 1. Introduction

The power cable is a critical component in building power systems, and any failure could potentially jeopardize the safety of the power system, leading to significant economic losses [1,2,3]. The insulation level of XLPE cables is closely linked to partial discharges, with the number of partial discharges serving as a characterization of the insulation condition of XLPE cables [4]. Many domestic and international researchers, as well as prominent power organizations such as IEEE and CIGRE, consider partial discharge detection as the most effective method for assessing the insulation condition of XLPE cables [5].

Recently, some scholars have researched the pattern recognition of partial discharge in power cables. For instance, Lu Chuang et al. studied the main insulation cuts, core burrs, residual metal particles, and outer sealing ruptures as four typical defects of XLPE cables. They utilized the FPGA acquisition card for partial discharge data sampling and selected skewness, steepness, and the number of inter-relationships as identifying features, using the support vector machine algorithm for classification [6]. Li Rong et al. classified typical defects of the cable body into capacitive and inductive categories, proposing a method for identifying and localizing local defect types in power cable bodies based on the input impedance spectrum [7]. Zhu Yufeng et al. used CNN (convolutional neural network) to identify the partial discharge signals of four types of insulation defects: core tip burrs, insulation internal air gaps, insulation surface scratches, and creepage of the outer semiconducting layer [8]. However, there are limited studies on partial discharge identification for composite defect cable models. Considering the multiple factors causing cable defects, it is essential to research partial discharge classification and identification for composite defect cable models.

In light of this, the paper designs and trains the Snake Optimizer–Support Vector Machine for four types of composite defect models of cables. The objective is to perform pattern recognition for partial discharge signals associated with these four composite defects using the SO-SVM model. Additionally, traditional learning models such as BP neural network, GA-BP neural network, and support vector machine (SVM) are used for the pattern recognition of partial discharge signals from cables. The pattern recognition effect of the SO-SVM model is compared and analyzed with the recognition effect of three traditional learning models, providing data reference and theoretical support for the pattern recognition of partial discharge defect types in XLPE cables.

## 2. Partial Discharge Experiment of XLPE Cable Insulation Defects Based on the High-Frequency Current Method

The high-frequency current method, an improvement on the pulse current method of foreign researchers, is currently the most widely applied technique in practical work [9,10]. This method avoids direct connection between high-frequency current sensors and high-voltage electrical equipment; its principle involves coupling cable partial discharge signals by installing a high-frequency current sensor around the grounding wire of the power cable’s copper shield [11]. When partial discharges occur within the cable insulation, the resulting high-frequency pulses of the partial discharge current travel along the cable’s copper shield grounding wire to ground. Consequently, fitting the high-frequency current sensor around the grounding wire of the cable’s copper shield enables the sensing of these locally generated discharge currents. The approach involves coupling detection of discharge pulse current signals passing through the cable grounding wire or the cable itself, making it suitable for situations where the cable shield is grounded [12]. This method boasts significant advantages in terms of implementation and test results: the detection device is easy to install and portable, the testing procedure is straightforward, it has a broad frequency range for detecting partial discharge pulse signals, and it offers strong anti-interference capabilities. Furthermore, it allows for flexible adjustment based on actual conditions and specific requirements, thus providing high adaptability [13].

### 2.1. Partial Discharge Experiment Platform Construction

**Figure 1:**
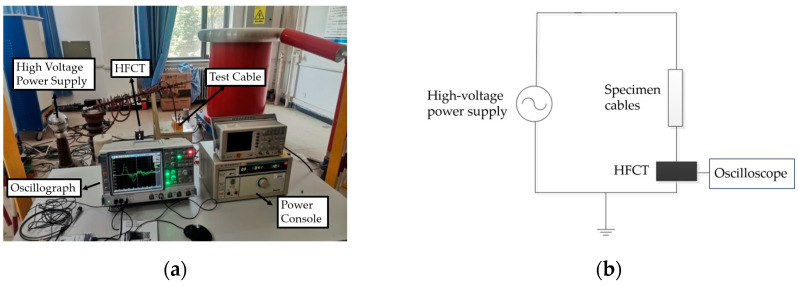
(**a**) Physical wiring diagram of the high-frequency current partial discharge test platform; (**b**) schematic diagram of partial discharge detection by the high-frequency current method.

A physical wiring diagram and electrical schematic of the partial discharge experimental platform are depicted in Figure 1. The setup includes the CS2674C high-voltage power supply from Nanjing Changsheng Instrument Co., Ltd. (Nanjing, China), the Rohde & Schwarz RTE1104 oscilloscope, a cross-linked polyethylene cable specimen, and a high-frequency current transducer, among other components. The high-voltage power supply offers a voltage test range of 3–50 kV and a leakage current test range of 0.5–20 mA. Additionally, the industrial frequency test transformer has a capacity of 1500 VA. The oscilloscope is capable of capturing 1 million waveforms per second, with a bandwidth of up to 2 GHz and a memory depth of 50 MSa per channel. The high-frequency current sensor adopted is the TWHCT-8033K model from Baoding Tianwei Xinyu Technology. This sensor has a detection frequency range of 100 kHz to 30 MHz, a transmission impedance greater than 15 mV/mA, and a detection sensitivity of 1 pc.

### 2.2. Design of a Four-Category Composite Defect Model for Cables

**Figure 2:**
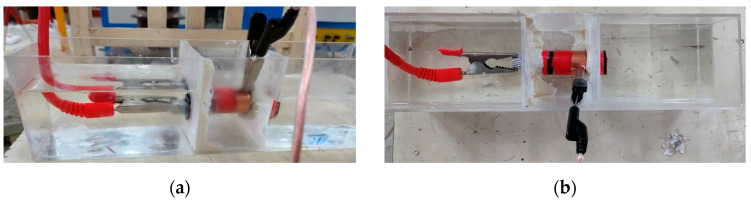
(**a**) Front view of cable defect partial discharge test device; (**b**) top view of cable defect partial discharge test device.

Firstly, a insulation scratch defect was artificially created on the XLPE cable. This was achieved by inserting a 1 mm diameter steel needle into the main insulation of the cross-linked polyethylene to a depth of 1.5 cm and then retracting it, thereby simulating a primary insulation scratch defect model. However, due to the exposure of cable ends to air, they were highly susceptible to surface discharges, rendering it challenging to accurately measure the partial discharge signals of the artificially created defects. Consequently, an experimental apparatus for detecting partial discharge signals in cables, based on insulating oil, was developed. This involved the use of acrylic material to construct a rectangular box with two round holes in the partition at its center, thereby dividing the box into three main compartments. The cable samples were placed in the round holes of the two central dividers, and the apertures were sealed with butyl waterproof adhesive. Insulating oil was then injected at both ends of the box, immersing the cable ends in the oil to prevent surface discharges from occurring. The insulating oil was replaced before each experiment to ensure its superior quality. The setup is illustrated in Figure 2.

**Figure 3:**
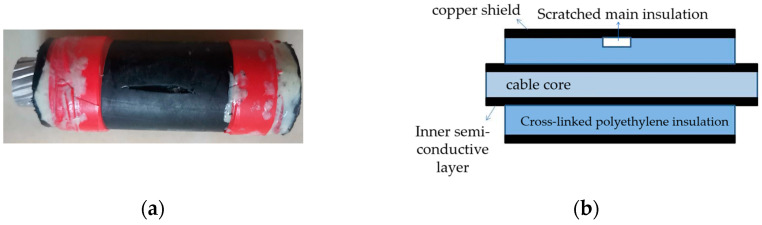
(**a**) Physical picture of scratch defect on main cable insulation; (**b**) model of scratch defect on main cable insulation.

**Figure 4:**
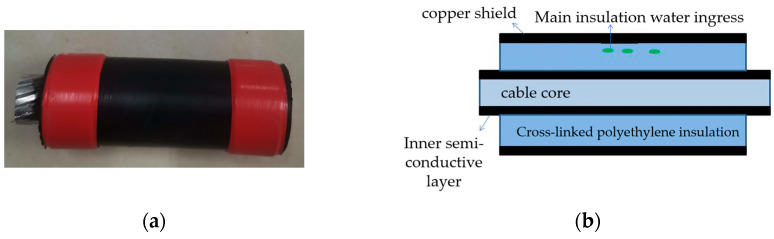
(**a**) Physical picture of water inlet defect on main cable insulation; (**b**) model of water inlet defect on main cable insulation.

**Figure 5:**
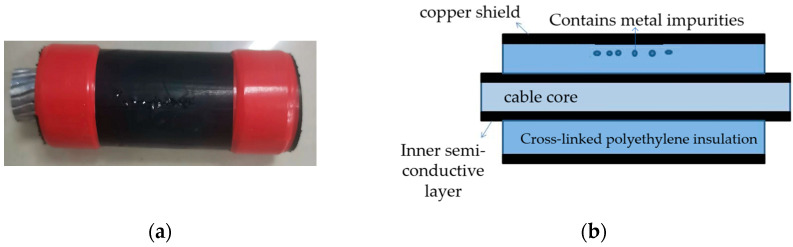
(**a**) Physical picture of metal impurity defect on main cable insulation; (**b**) model of metal impurity defect on main cable insulation.

The three-core XLPE cables were separated and transformed into single-core cables to replicate three typical insulation defects: scratches on the main insulation, water ingress in the main insulation, and the presence of metallic contaminants. To simulate the main insulation scratch defects (phase A), a section of the cross-linked polyethylene main insulation within the copper shield of the cable sample was excavated. A schematic diagram of the specimen cable is illustrated in Figure 3. Subsequently, the cable samples were submerged in water for 48 h to replicate the main insulation water ingress defects (phase B), as depicted in the schematic diagram in Figure 4. Furthermore, several metallic iron particles, approximately 1 mm in diameter, were embedded within the cross-linked polyethylene’s main insulation to model defects involving metallic impurities (phase C), as shown in the schematic diagram in Figure 5. These three types of typical defects were then combined to create four composite defect models, including scratches and water ingress; metal-containing contaminants and scratches; metal-containing contaminants and water ingress; and metal-containing contaminants, water ingress, and scratches.

### 2.3. Detection of Partial Discharge Signals for Four Types of Composite Defects

A test setup utilizing insulating oil was employed to examine the partial discharge signals emitted from a fabricated single-core cable model incorporating compound defects. In the course of the experiment, the cable was meticulously enveloped with soft copper foils to function as a shielding layer. The cable was situated within the circular apertures of two dividing panels in the center of the inspection apparatus, with the joints around these apertures sealed using butyl water-resistant adhesive. Subsequently, insulating oil was infused into both ends of the container, thereby immersing the cable’s extremities in the oil. Thereafter, the copper shielding layer of the cable was grounded. Application of high voltage to the cable facilitated the acquisition of partial discharge signals. When partial discharge pulse signals appeared on the oscilloscope, the voltage applied to the specimen cable by the high-voltage power supply was as shown in Table 1.

**Figure 6:**
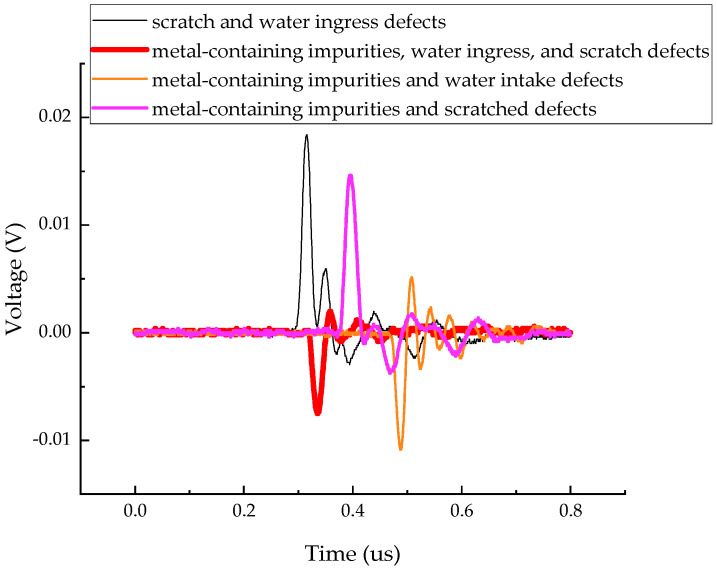
The partial discharge signals for four types of composite defects.

The measured partial discharge signals for four types of composite defects are shown in Figure 6.

## 3. Partial Discharge Signal Feature Extraction Based on VMD and Entropy Value

Dragomiretskiy and Zosso proposed the Variational Mode Decomposition (VMD) in 2014, which is a scale-adaptive method for processing non-stationary signals [14]. VMD effectively decomposes a complex signal into multiple intrinsic mode components and a residual component, thereby fundamentally resolving the problems of mode mixing and endpoint effects inherent in Empirical Mode Decomposition (EMD) [15]. The VMD algorithm primarily comprises two aspects: the formulation of the variational problem and its subsequent solution [16].

### 3.1. Eigenvalue Extraction Based on VMD and Power Spectral Entropy

**Figure 7:**
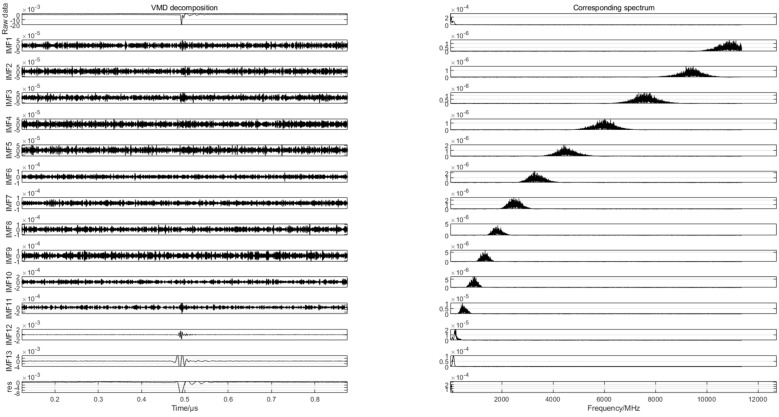
VMD decomposition of partial discharge signals for scratches and water inlet defects.

**Figure 8:**
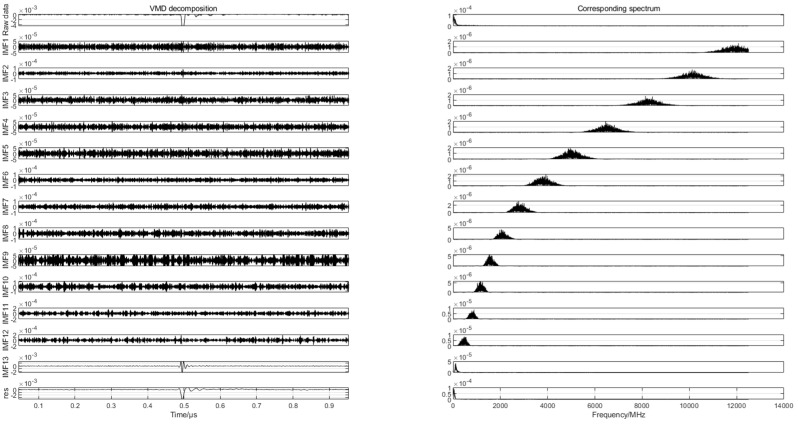
VMD decomposition of partial discharge signals containing metal impurities and scratch defects.

**Figure 9:**
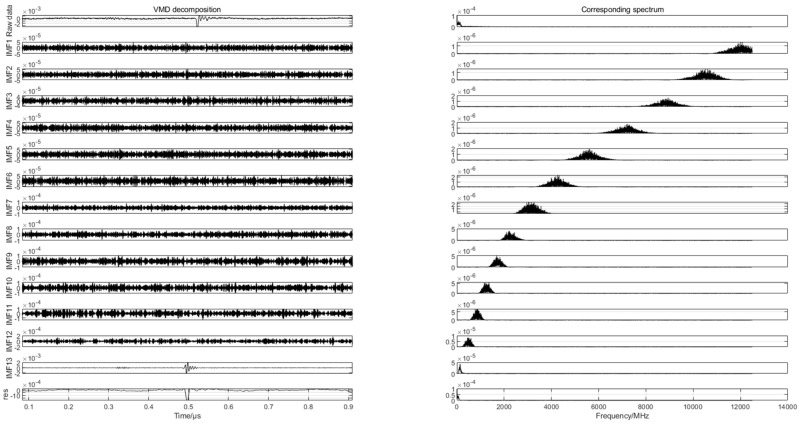
VMD decomposition of partial discharge signals containing metal impurities and influent defects.

**Figure 10:**
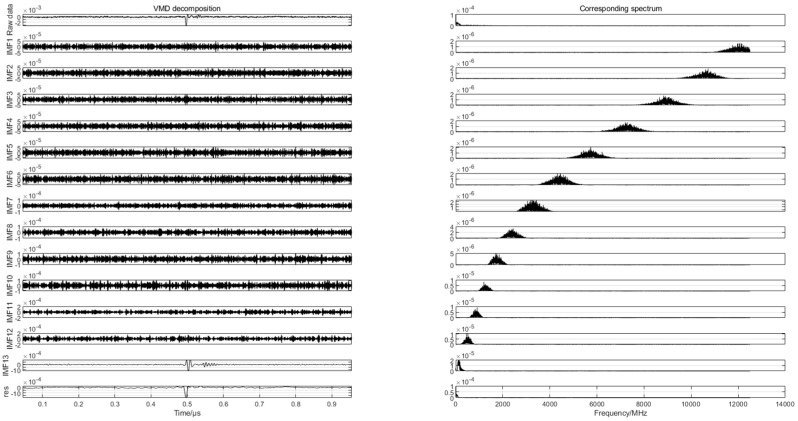
VMD decomposition of partial discharge signals containing metal impurities and water intake and scratch defects.

To find the differences in the partial discharge signals of the four types of composite defects, the differences are used for the pattern recognition of the partial discharge signals of the four types of composite defects. In this paper, 20 samples are randomly selected from the partial discharge signals of four types of composite defects collected from the partial discharge experiments for variational mode decomposition. Each raw partial discharge signal is decomposed into 13 modal components and a residual component. The VMD decompositions of each of the partial discharge signals of the four types of composite defects and their corresponding spectra are shown in Figure 7, Figure 8, Figure 9 and Figure 10.

**Figure 11:**
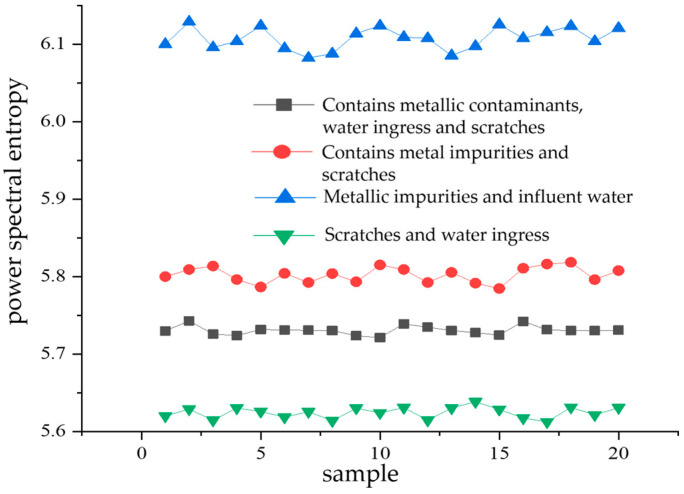
Comparison of the mean power spectrum entropy of each mode component of four types of compound defect partial discharge signals.

The principle of power spectral analysis is that the total energy contained within a signal is equal to the sum of the energies of its individual components across a complete set of orthogonal functions [17,18]. In this paper, 20 sample datapoints are randomly selected for VMD decomposition for each kind of composite defect partial discharge signal. Then, the entropy value of the power spectrum of each modal component obtained from decomposition is calculated. Then, the average of the power spectral entropy of the 13 modal components of each partial discharge signal is obtained (PS). A comparison of the mean PS of the power spectral entropy of each modal component calculated from 20 samples of partial discharge signals for each of the four types of composite defects is shown in Figure 11.

From Figure 11, it can be seen that the mean value of power spectral entropy of the partial discharge signals containing metal impurities and water intake defects is the greatest. Its value fluctuates around 6.1, indicating that among the four types of composite defects, the energy distribution of the partial discharge signals of metal-containing impurities and water-entry defects is the most complex in the frequency domain space. The lowest mean value of the power spectral entropy of partial discharge signals is obtained for scratch and water ingress defects. Its value fluctuates around 5.62, indicating that among the four types of composite defects, the partial discharge signals of scratch and water ingress defects have the lowest complexity of energy distribution in the frequency domain space.

Since there is a significant difference in the mean PS value of the power spectral entropy of the partial discharge signals of the four types of composite defects, this value can be used as an eigenvalue for subsequent pattern recognition.

### 3.2. Eigenvalue Extraction Based on VMD and Fuzzy Entropy

**Figure 12:**
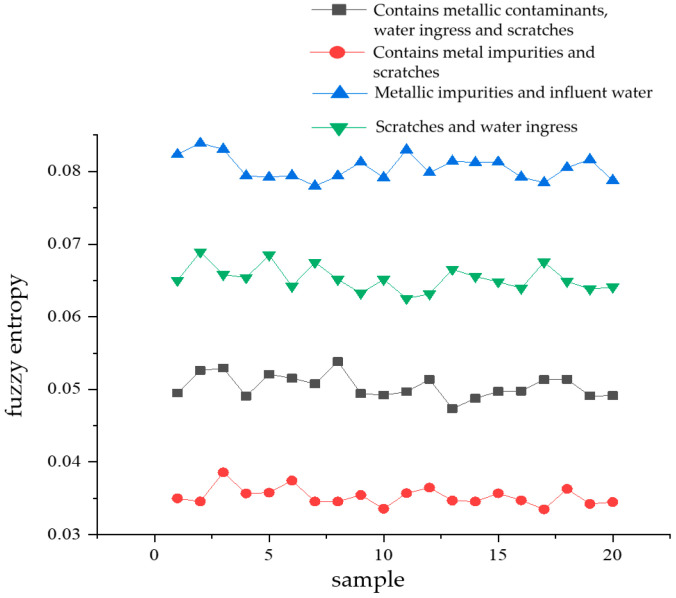
Comparison of fuzzy entropy mean values of each mode component of four types of compound defect partial discharge signals.

Fuzzy entropy characterizes the complexity of a time series; the simpler the time series, the smaller its fuzzy entropy value. Conversely, the more complex the series, the larger the fuzzy entropy value. Fuzzy entropy possesses a strong capability to resist interference from background noise [19,20,21]. For each type of complex defect, 20 sample datapoints of partial discharge signals were randomly selected and subjected to VMD decomposition. Then, the fuzzy entropy values of the various modal components obtained from the decomposition were calculated, followed by determining the average fuzzy entropy FU of the 13 modal components for each signal. Figure 12 illustrates a comparative diagram of the mean fuzzy entropy values (FU) for each modal component, computed from the 20 partial discharge signal samples of the four types of complex defects.

From Figure 12, it can be seen that the average value of fuzzy entropy (FU) of the partial discharge signals of metal-containing impurities and water intake defects is the largest. Its value fluctuates around 0.08, indicating that among the four types of composite defects in cables, the time series complexity of the partial discharge signals of defects containing metallic impurities and water ingress is the greatest. The lowest mean value of fuzzy entropy is obtained for partial discharge signals containing metallic impurities and scratched defects. Its value fluctuates around 0.035, indicating that among the four types of composite defects in cables, the time series complexity of the partial discharge signals of defects containing metallic impurities and scratches is the smallest. Since there is a significant difference in the magnitude of the fuzzy entropy mean value (FU) of the partial discharge signals of the four types of composite defects, the fuzzy entropy mean value (FU) can be used as an edge quantity for the subsequent pattern recognition.

### 3.3. Eigenvalue Extraction Based on VMD and Permutation Entropy

**Figure 13:**
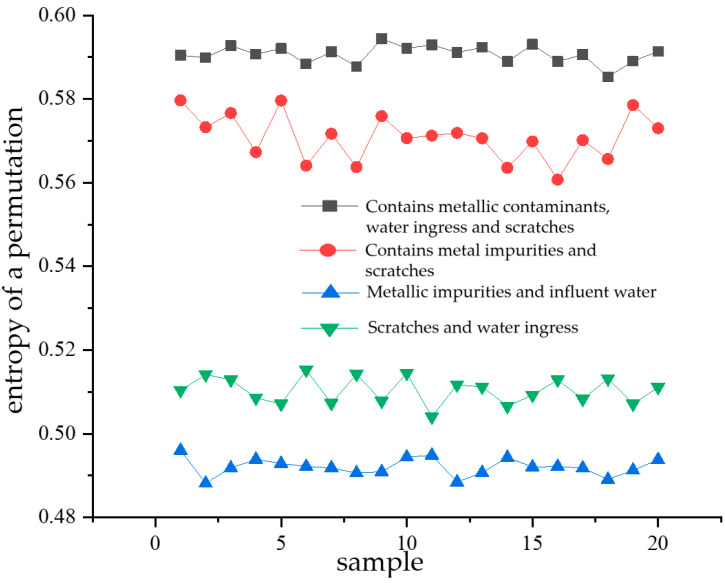
Comparison diagram of mean arrangement of entropy of each mode component of four types of compound defect partial discharge signals.

Permutation entropy, introduced by BANDT and POMPE, is used to quantify the degree of randomness in signal sequences. A smaller calculated permutation entropy value indicates that the signal sequence is more stable and regular, whereas a higher value suggests a stronger degree of randomness in the signal sequence [22,23]. Variational modal decomposition is performed for 20 randomly selected sample datapoints for each type of composite defect partial discharge signal, respectively. Then, the alignment entropy value of each modal component obtained from the decomposition is calculated, and then the average value of the alignment entropy of the 13 modal components of each partial discharge signal is obtained (PE). Shown in Figure 13 is a comparison of the mean PE of the entropy of each modal component alignment calculated from 20 samples of partial discharge signals for each of the four types of composite defects. From the figure, it can be seen that the average value of the entropy of the partial discharge signals of defects containing metal impurities, water ingress, and scratches is the largest, indicating that the randomness of the partial discharge signals of this type of defect is the strongest. The mean entropy value of the arrangement of the partial discharge signals of metal-containing impurities and water intake defects is the smallest, and its value fluctuates around 0.49. It indicates that among the four types of composite defects in cables, the partial discharge signal sequence of defects containing metallic impurities and water ingress is the smoothest and most regular. Since there is a significant difference in the magnitude of the average value of the arrangement entropy (PE) of the partial discharge signals of the four types of composite defects, the average value of the arrangement entropy (PE) can be used as an eigenvalue for the pattern recognition of the partial discharge signals of the four types of composite defects.

**Figure 14:**
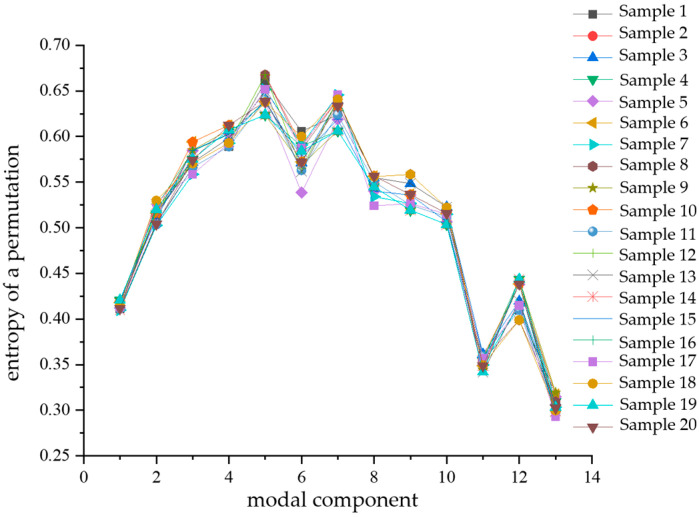
Scratches and water ingress.

**Figure 15:**
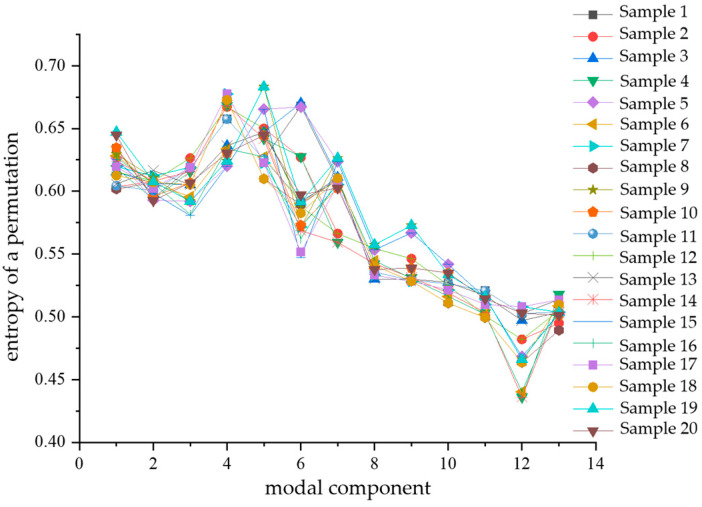
Contains metal impurities and scratches.

**Figure 16:**
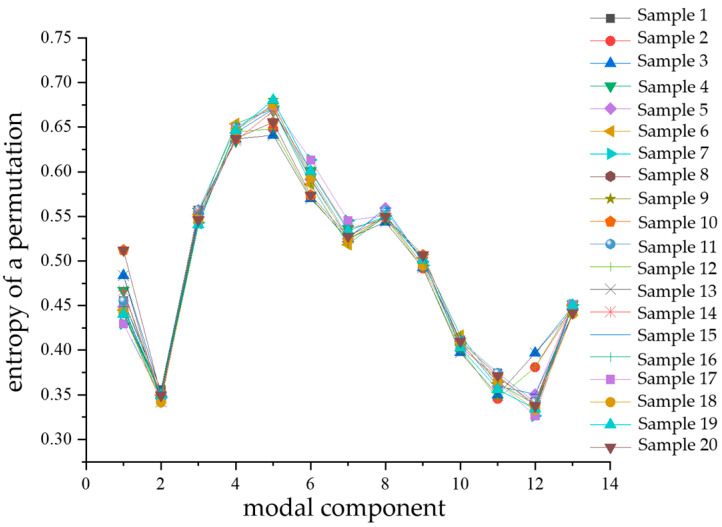
Contains metal impurities and water ingress.

**Figure 17:**
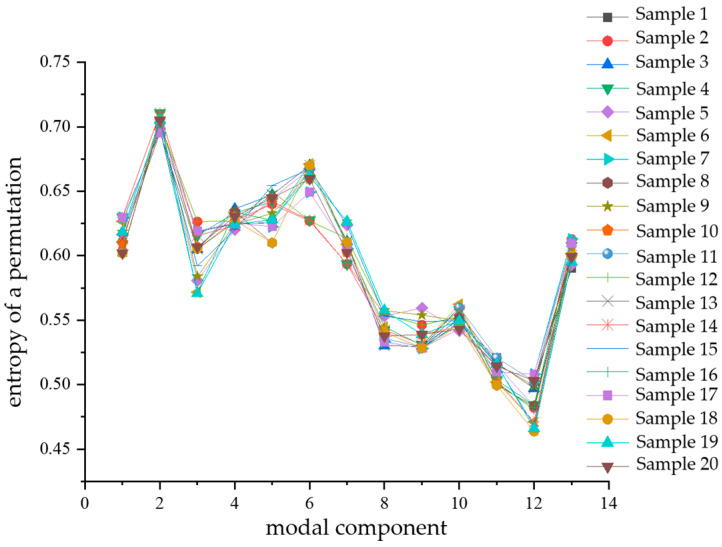
Contains metal impurities, water and scratch defects.

Similarly, after calculating the alignment entropy values of each modal component for a total of 80 samples of the four types of composite defects, a comparative analysis of the differences was performed with the modal components as the horizontal coordinates. The comparison diagrams are shown in Figure 14, Figure 15, Figure 16 and Figure 17. From the figures, it can be seen that there is a significant difference in the alignment entropy value Pe of the 2nd modal component IMF2 and the 13th modal component IMF13 for the four types of composite defects. The aligned entropy values of IMF2 for the four types of defects, scratches and water ingress; metal-containing contaminants and scratches; metal-containing contaminants and water ingress; and metal-containing contaminants, water ingress, and scratches, varied in the vicinity of 0.51, 0.6, 0.35, and 0.7, respectively. The value is the largest for defects containing metallic impurities, water ingress, and scratches, indicating that the IMF2 signal sequence of the partial discharge signals of such defects has the strongest randomness and the poorest regularity. This value is the smallest for IMF2 containing metal impurities and inlet defects partial discharge signals, indicating that the partial discharge signals of the IMF2 signal sequence containing metal impurities and inlet defect partial discharge signals has the weakest stochasticity, is the smoothest, and has the most regularity. The aligned entropy values of IMF13 for the four categories of defects, scratches, and water ingress; metal-containing contaminants and scratches; metal-containing contaminants and water ingress; and metal-containing contaminants, water ingress, and scratches, varied in the vicinity of 0.3, 0.5, 0.45, and 0.6, respectively. It indicates that among the four types of composite defects, the IMF13 signal sequence of partial discharge signals of defects containing metallic impurities, water ingress, and scratches has the strongest randomness and the worst regularity. The IMF13 signal sequence of partial discharge signals from scratches and water ingress defects has the weakest, smoothest, and most regular randomness. Since these two values vary in different value ranges, respectively, the arrangement entropy values (Pe) of IMF2 and IMF13 can be used as two eigenvalues for subsequent pattern recognition.

Consequently, five feature values are selected for composite defect partial discharge signals: the mean power spectrum entropy of modal components (PS), the mean fuzzy entropy (FU), the mean permutation entropy (PE), and the permutation entropy values of IMF2 and IMF13 (Pe). A total of 600 samples are taken from each type of composite defect partial discharge signal, totaling 2400 samples for the four defect types. Each sample comprises five feature values, which are compiled into a dataset. Of these, 80% (1920 samples) are allocated to the training dataset, while 20% (480 samples) constitute the testing dataset. The classification of the four types of composite defect partial discharge signals in cables is then performed using the SO-SVM model in conjunction with traditional learning models.

## 4. Pattern Recognition of Partial Discharge Signals from Cable Insulation Defects Based on SO-SVM

### 4.1. Snake Optimizer–Support Vector Machine

With the advancement of artificial intelligence, time series forecasting has gradually come into the spotlight and gained significant attention. The application of support vector machine (SVM) is becoming increasingly prevalent. SVM is a supervised machine learning model capable of fitting nonlinear data and demonstrating robust tolerance to noise and outliers. Its fundamental principle revolves around identifying an optimal hyperplane that maximally separates samples of different classes. This approach allowed SVMs to perform excellent generalization, even with small training datasets [24]. However, The performance of SVM depends mainly on the selection of model parameters [25]. When employing SVM for time series prediction, it is necessary to tune parameters to achieve the best forecasting performance. Conventional parameter tuning algorithms often entail substantial computational time and may run the risk of becoming stuck in local optima.

The Snake Optimizer (SO), inspired by the foraging and reproduction behaviors of snakes, was proposed in 2022 by professors Hashim, F. A., and Hussien, A. G. [26].

The SO algorithm is capable of searching for the optimal solution in the parameter space, characterized by fast convergence speed and strong global search capability, making it highly effective for application in parameter optimization problems.

The SO-SVM model incorporates the Snake Optimization Algorithm into the traditional SVM framework, iteratively optimizing SVM’s penalty parameter (c) and kernel function parameter (g) to enhance classification accuracy. The following are the steps for optimizing support vector machine using the Snake Optimization Algorithm:

1. Firstly, determine the input and output of the fault diagnosis model, establish the training set and test set, and input the feature dimensions;
2. Set variables to store data, and normalize the training set and test set to the [0, 1] interval;
3. Define the number of optimization parameters. In this scenario, the number of optimization parameters is 2 (c, g);
4. SO parameter settings: set the population size and maximum number of iterations;
5. Use SO optimization to obtain c and g, and conduct training and testing;
6. Compare the actual values with the predicted values, and output the accuracy rate.

### 4.2. Recognition and Result Analysis of Partial Discharge Signals Based on SO-SVM

#### Evaluation of the Recognition Performance of SO-SVM

**Figure 18:**
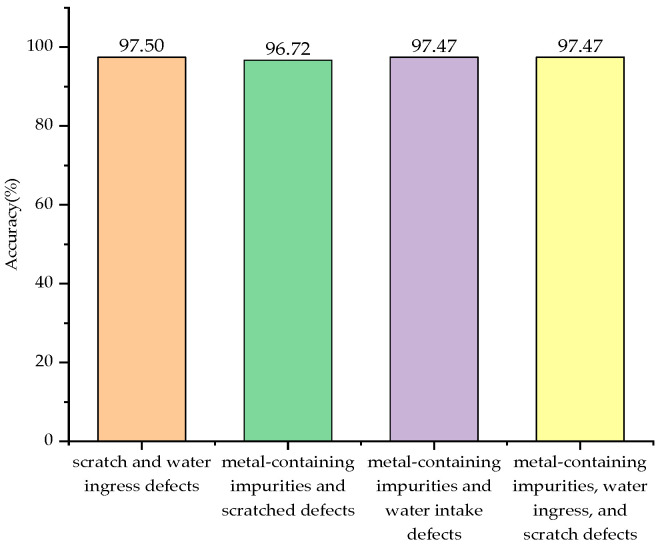
Identification accuracy of four types of compound defect partial discharge signals.

The pattern recognition of four types of complex defect partial discharge signals was conducted using SO-SVM, and the results are shown in Table 2. As can be seen from Table 2, the SO-SVM classifier successfully identified 117 samples with scratch and water ingress defects, 118 samples with metal impurity and scratch defects, 116 samples with metal impurity and water ingress defects, and 116 samples with metal impurities, water ingress, and scratch defects among the 480 test samples. In total, 467 samples were correctly identified out of 480, achieving an identification success rate of 97.29%. The accuracy comparison for identifying partial discharge signals of the four complex defect types is illustrated in Figure 18.

**Figure 19:**
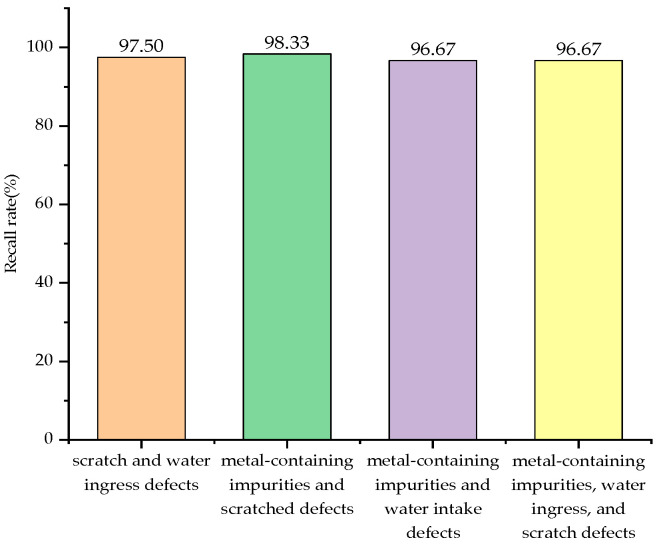
Recall rate of four kinds of compound defect partial discharge signals.

**Figure 20:**
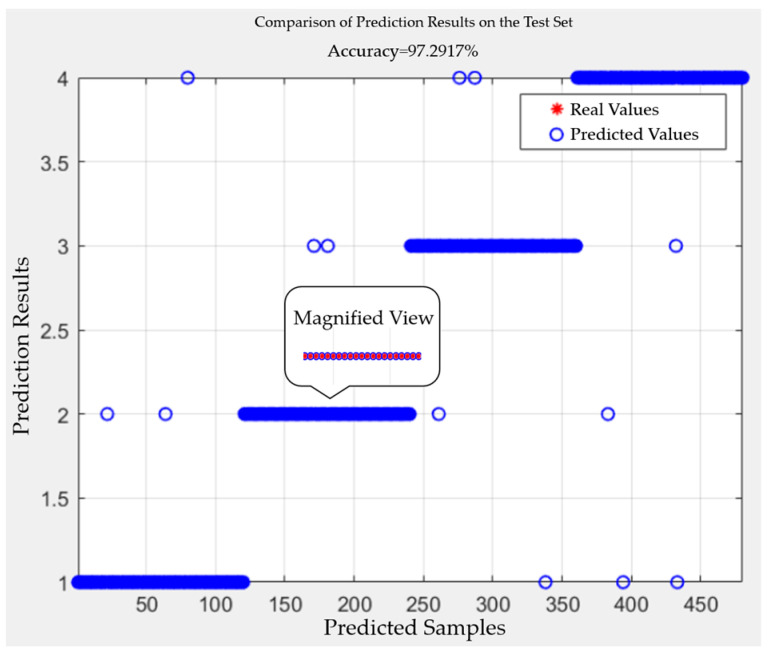
SO-SVM classification effect diagram.

Recall rate, also known as the detection rate, is a measure of coverage that can quantify how many signals are accurately identified. A comparison chart of the recall rates for the four types of composite defect partial discharge signals is shown in Figure 19. From the figure, it is intuitively clear that the recall rates for all four types of composite defect partial discharge signals are quite high, indicating that a significant number of signals are accurately predicted. The classification effect of SO-SVM is illustrated in Figure 20.

## 5. Pattern Recognition of Partial Discharge Signals Based on Conventional Learning Models

### 5.1. Pattern Recognition of Partial Discharge Signal Based on BP Neural Network and SVM

The BP neural network model classifier successfully identified a total of 86 samples with scratch and water ingress defects, 85 samples with metallic contaminant and scratch defects, 82 samples with metallic contaminant and water ingress defects, and 83 samples with metallic contaminant, water ingress, and scratch defects, out of a total of 400 samples in the test dataset. Overall, a total of 336 out of 400 samples were successfully recognized, with a recognition success rate of 84.00%. An analysis of Table 3 shows that the identification accuracies of the four types of composite defects, namely, scratches and water ingress; metal-containing contaminants and scratches; metal-containing contaminants and water ingress; and metal-containing contaminants, water ingress, and scratches, are 83.50%, 83.33%, 83.67%, and 85.57%, respectively.

In addition, based on the collected partial discharge signals of four types of composite defects in XLPE cables, 600 sets of data were extracted from each type of defective partial discharge signal. A random selection of 200 of these groups were used to train and learn the SVM classifier model, and the remaining 400 groups were used as a test dataset to test the effectiveness of pattern recognition. The results are shown in Table 4. From Table 4, we can see that the recognition accuracies of the four types of composite defects, namely, scratches and water ingress; metal-containing contaminants and scratches; metal-containing contaminants and water ingress; and metal-containing contaminants, water ingress, and scratches, are 85.00%, 84.16%, 83.50%, and 85.42%, respectively.

### 5.2. GA-BP-Based Pattern Recognition of Partial Discharge Signals

Based on the collected partial discharge signals of four types of composite defects of XLPE cables, the GA-BP classifier model was used for the pattern recognition of partial discharge signals of four types of composite defects of cables. The results are shown in Table 5. From Table 5, it can be obtained that the identification accuracies of the four types of composite defects, namely, scratches and water ingress; metal-containing contaminants and scratches; metal-containing contaminants and water ingress; and metal-containing contaminants, water ingress, and scratches, were 91.84%, 87.38%, 88.00%, and 87.88%, respectively.

### 5.3. Comparison of Recognition Effectiveness between the SO-SVM Model and Traditional Learning Model

This paper compares the pattern recognition capabilities of the SO-SVM model with traditional learning models such as BP neural networks, GA-BP neural networks, and SVM for four types of composite defect partial discharge signals. Table 6 shows the recognition accuracy of the four types of composite defect partial discharge signals for each model. As can be seen from Table 6, the SO-SVM model has the highest recognition accuracy for partial discharge signals compared to other models. For scratch and water ingress defects, SO-SVM improved by 14.00% compared to BP neural networks, 5.66% compared to GA-BP, and 12.50% compared to SVM. For metal impurities and scratch defects, SO-SVM improved by 13.39% compared to BP, 9.34% compared to GA-BP, and 12.56% compared to SVM. For metal impurities and water ingress defects, SO-SVM improved by 13.80% compared to BP, 9.47% compared to GA-BP, and 13.97% compared to SVM. For metal impurities, water ingress, and scratch defects, SO-SVM improved by 11.90% compared to BP, 9.59% compared to GA-BP, and 12.05% compared to SVM. 

**Figure 21:**
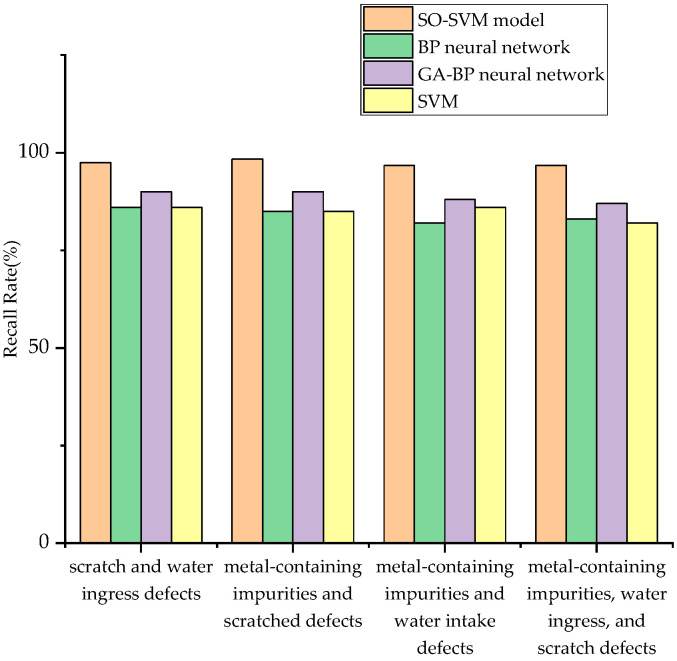
Comparison of partial discharge recall rates of four types of compound defects by different model classifiers.

A comparison of the partial discharge signal recall of the four types of composite defects for various model classifiers is shown in Figure 21. From the figure, it can be visualized that the SO-SVM model has the highest recall for the partial discharge signals of the four types of composite defects, indicating that the method is the most accurate for the identification of sample data.

## 6. Conclusions

This paper presents the development of an experimental platform for detecting partial discharge signals of 10 kV XLPE cables based on the high-frequency current method. An experimental device utilizing insulating oil is designed to detect the partial discharge signals of cables, aiming to prevent cable end discharges along the surface and achieve the detection of partial discharge signals associated with four types of composite defects. Subsequently, the entropy values of power spectral entropy, fuzzy entropy, and arrangement entropy of each modal component are calculated based on the variational modal decomposition of the acquired partial discharge signals, followed by variability analysis. Five types of feature quantities are obtained, and the SO-SVM model is employed along with three traditional learning models for the pattern recognition of partial discharge signals. Finally, the recognition effect of the SO-SVM model is compared with that of the traditional learning models, leading to the following conclusions:

1. An experimental device for partial discharge signal detection in cables based on insulating oil is proposed, which can prevent the occurrence of surface discharge along the ends of the test cables and accurately collect the partial discharge signals of artificially set defects.
2. Significant differences are observed in the mean power spectral entropy, mean fuzzy entropy, and mean permutation entropy of the modal components obtained after VMD decomposition of the partial discharge signals from four types of composite defects. There is also a notable difference in the alignment entropy values of the modal components IMF2 and IMF13. These findings suggest that the detected partial discharge signals can be distinguished by their eigenvalues.
3. The SO-SVM model outperforms traditional learning models in recognizing PD signals from composite insulation defects in XLPE cables. In terms of recognition accuracy, for scratch and water ingress defects, SO-SVM achieves an improvement of 14.00% over the BP neural network, 5.66% over GA-BP, and 12.50% over SVM. For defects involving metal impurities and scratches, SO-SVM improves by 13.39% compared to BP, 9.34% compared to GA-BP, and 12.56% compared to SVM. In the case of metal impurities and water ingress defects, SO-SVM shows an improvement of 13.80% over BP, 9.47% over GA-BP, and 13.97% over SVM. Finally, for defects combining metal impurities, water ingress, and scratches, SO-SVM achieves an improvement of 11.90% over BP, 9.59% over GA-BP, and 12.05% over SVM.

## Figures and Tables

**Table 1:** The voltages at which partial discharge pulse signals occur for four types of composite defect models.

Defects	Scratches and Water Ingress	Contains Metal Impurities and Scratches	Metallic Impurities and Influent Water	Contains Metallic Contaminants, Water Ingress, and Scratches
The voltage at which partial discharge pulse signals occur	4.5 kV	5.1 kV	4.6 kV	2.9 kV

**Table 2:** Results of partial discharge pattern recognition for four types of composite defects based on SO-SVM.

	Identify Defects	Scratches and Water Ingress	Contains Metal Impurities and Scratches	Metallic Impurities and Influent Water	Contains Metallic Contaminants, Water Ingress, and Scratches	Total Sample Size
Genuine Defects						
Scratches and water ingress		117	2	0	1	120
Contains metal impurities and scratches		0	118	2	0	120
Metallic impurities and influent water		1	1	116	2	120
Contains metallic contaminants, water ingress, and scratches		2	1	1	116	120
Total defects identified		120	122	119	119	480

**Table 3:** Recognition results of four types of compound defect partial discharge patterns based on BP neural network.

	Identify Defects	Scratches and Water Ingress	Contains Metal Impurities and Scratches	Metallic Impurities and Influent Water	Contains Metallic Contaminants, Water Ingress and Scratches	Total Sample Size
Genuine Defects						
Scratches and water ingress		86	6	3	5	100
Contains metal impurities and scratches		5	85	7	3	100
Metallic impurities and influent water		7	5	82	6	100
Contains metallic contaminants, water ingress and scratches		5	6	6	83	100
Total defects identified		103	102	98	97	400

**Table 4:** Results of four types of compound defect partial discharge pattern recognition based on the SVM classifier model.

	Identify Defects	Scratches and Water Ingress	Contains Metal Impurities and Scratches	Metallic Impurities and Influent Water	Contains Metallic Contaminants, Water Ingress, and Scratches	Total Sample Size
Genuine Defects						
Scratches and water ingress		86	6	6	3	100
Contains metal impurities and scratches		5	85	4	6	100
Metallic impurities and influent water		6	3	86	5	100
Contains metallic contaminants, water ingress, and scratches		4	7	7	82	100
Total defects identified		100	101	103	96	400

**Table 5:** Recognition results of four types of compound defect partial discharge patterns based on GA-BP.

	Identify Defects	Scratches and Water Ingress	Contains Metal Impurities and Scratches	Metallic Impurities and Influent Water	Contains Metallic Contaminants, Water Ingress and Scratches	Total Sample Size
Genuine Defects						
Scratches and water ingress		90	2	3	5	100
Contains metal impurities and scratches		3	90	4	3	100
Metallic impurities and influent water		3	5	88	4	100
Contains metallic contaminants, water ingress, and scratches		2	6	5	87	100
Total defects identified		98	103	100	99	400

**Table 6:** Identification accuracy of four types of compound defect partial discharges by different model classifiers.

	Recognition Accuracy	Scratches and Water Ingress	Contains Metal Impurities and Scratches	Metallic Impurities and Influent Water	Contains Metallic Contaminants, Water Ingress and Scratches	Average Recognition Accuracy
Model						
SO-SVM model		97.50%	96.72%	97.47%	97.47%	97.29%
BP neural network		83.50%	83.33%	83.67%	85.57%	84.02%
GA-BP neural network		91.84%	87.38%	88.00%	87.88%	88.78%
SVM		85.00%	84.16%	83.50%	85.42%	84.52%

## Data Availability

Data are contained within the article.
